# CDK4/6 inhibition is more active against the glioblastoma proneural subtype

**DOI:** 10.18632/oncotarget.19429

**Published:** 2017-07-21

**Authors:** Ming Li, Aizhen Xiao, Desiree Floyd, Inan Olmez, Jeongwu Lee, Jakub Godlewski, Agnieszka Bronisz, Krishna P.L. Bhat, Erik P. Sulman, Ichiro Nakano, Benjamin Purow

**Affiliations:** ^1^ Neuro-Oncology Division, Department of Neurology, University of Virginia, Charlottesville, VA, USA; ^2^ The Experiment Center, The Second Affiliated Hospital of Soochow University, Suzhou, Jiangsu, China; ^3^ Department of Stem Cell Biology and Regenerative Medicine, Lerner Research Institute, Cleveland Clinic, Cleveland, OH, USA; ^4^ Department of Neurosurgery, Brigham and Women's Hospital, Boston, MA, USA; ^5^ Departments of Pathology, MD Anderson Cancer Center, Houston, TX, USA; ^6^ Radiation Oncology, MD Anderson Cancer Center, Houston, TX, USA; ^7^ Department of Neurosurgery, University of Alabama, Birmingham, AL, USA

**Keywords:** glioblastoma, palbociclib, proneural, mesenchymal, CDK4/6

## Abstract

Glioblastoma (GBM) is the most common and lethal brain tumor. Gene expression profiling has classified GBM into distinct subtypes, including proneural, mesenchymal, and classical, and identifying therapeutic vulnerabilities of these subtypes is an extremely high priority. We leveraged The Cancer Genome Atlas (TCGA) data, in particular for microRNA expression, to seek druggable core pathways in GBM. The E2F1-regulated miR-17˜92 cluster and its analogs are shown to be highly expressed in proneural GBM and in GSC lines, suggesting the E2F cell cycle pathway might be a key driver in proneural GBM. Consistently, CDK4/6 inhibition with palbociclib preferentially inhibited cell proliferation *in vitro* in a majority of proneural GSCs versus those of other subtypes. Palbociclib treatment significantly prolonged survival of mice with established intracranial xenografts of a proneural GSC line. We show that most of these sensitive PN GSCs expressed higher levels of CDK6 and had intact Rb1, while two GSC lines with CDK4 overexpression and null Rb1 were highly resistant to palbociclib. Importantly, palbociclib treatment of proneural GSCs upregulated mesenchymal-associated markers and downregulated proneural-associated markers, suggesting that CDK4/6 inhibition induced proneural-mesenchymal transition and underscoring the enhanced role of the E2F cell cycle pathway in the proneural subtype. Lastly, the combination of palbociclib and N,N-diethylaminobenzaldehyde, an inhibitor of the mesenchymal driver ALDH1A3, showed strong synergistic inhibitory effects against proneural GSC proliferation. Taken together, our results reveal that proneural GBM has increased vulnerability to CDK4/6 inhibition, and the proneural subtype undergoes dynamic reprogramming upon palbociclib treatment—suggesting the need for a combination therapeutic strategy.

## INTRODUCTION

Glioblastoma (GBM) is the most common and most lethal primary brain tumor, causing 12–14,000 deaths each year in the U.S. alone [1]. Median survival following diagnosis is approximately 12–15 months with current therapy including maximal surgical resection, radiation, and temozolomide chemotherapy [2]. While all GBMs share histopathological and clinical features, The Cancer Genome Atlas (TCGA) and other profiling efforts have revealed two to four GBM subtypes: proneural (PN) and mesenchymal (MES) have been most reliably established, with classical (CL) and neural subtypes also described [3, 4]. PN subtype typically arises in frontal cortex and often has *PDGFRA* amplification, *IDH1*/*IDH2* mutation, and *TP53* mutations; those with *IDH1*/*IDH2* mutation—which includes most secondary GBMs arising from low-grade gliomas—have the best prognosis of any GBM subgroup, but proneural GBM without IDH mutations have perhaps the worst outcomes [3–5]. MES subtype is aggressive and has greater vascularity, and it has been associated with *NF1* lesions and with higher Akt, TGF-β, and NF-κB activity [3–5]. CL subtype is also aggressive and is marked by frequent *EGFR* lesions [3–5]. The neural subtype has become controversial, as it is less distinct and may arise from substantial contamination of GBM samples with normal brain [5]. Major efforts have been underway to identify critical drivers of each GBM subtype, in hopes of gaining therapeutic leverage against them. Unfortunately, little progress has been made in uncovering key driver pathways and therapeutic vulnerabilities of the GBM subtypes, other than a few reports suggesting core circuitry of the MES subtype [6–8].

microRNAs (miRNAs) are endogenous 20–22 bp small RNAs that do not encode peptides, but powerfully regulate gene expression by blocking translation or impairing RNA stability of mRNAs with 3′-untranslated regions (3′-UTRs) containing target sites for those miRNAs [9]. Numerous miRNAs are dysregulated in cancer and play oncogenic or tumor-suppressive roles. The majority of over- and under-expressed miRNAs in cancer are likely dysregulated due to aberrant activity of oncogenic or tumor-suppressive pathways that regulate their expression. This suggests that signatures of dysregulated miRNAs may shed light on the core driver pathways within cancers.

To identify novel potential drivers in GBM subtypes, we performed *in silico* analysis of TCGA data and found that several in the miR-17˜92 cluster or in analogous clusters are highly upregulated in the PN subtype of GBMs, and these are known to be transcriptionally up-regulated by the E2F cell cycle and myc pathways [10–12]. E2F drives cell cycle entry, and its activity is regulated by inputs from activators (CDK and cyclin proteins) and inhibitors (such as phosphorylated/activated Rb1 and the INK4 family p15^INK4b^, p16^INK4a^, p18^INK4c^, and p19^INK4d^). The work published by TCGA has revealed that the CDK4/6-Rb-E2F pathway is frequently disrupted in 78% of GBMs. The most common alteration of this pathway is homozygous deletion of p15 and p16, which is present in 50% of tumors, as well as mutations in *CDKN2A*. Amplification/overexpression of *CDK4* is detected in 15–20% of GBM, while homozygous deletion/mutation of *RB1* is present in 7.6% of GBM. Amplification of *CDK6* and homozygous deletion of *p18* are less common (2%) [5, 13]. Our TCGA microRNA findings, in addition to the frequent dysregulation of the E2F cell cycle pathway in GBM, led us to suspect the prominent involvement of this pathway in PN GBM and that it might represent a therapeutic vulnerability.

To further investigate whether the E2F cell cycle pathway was especially relevant in proneural GBM, we utilized palbociclib, a first-generation inhibitor of CDK4/6 recently approved by the FDA for breast cancer and now in clinical trials for patients with GBM [14, 15]. In this study, we tested the relative sensitivity of a panel of GBM stem cell-like (GSC) lines to palbociclib, finding that it preferentially inhibits cell proliferation and induces G1 phase arrest in PN GSC lines with high expression of CDK6 and functional Rb1. Moreover, we show that CDK4/6 inhibition may induce PN-MES transition in GSCs, and that targeting both CDK4/6 and a MES driver synergistically inhibits proneural GSC proliferation.

## RESULTS

### The miR-17˜92 family and its paralogs are elevated in human PN GBM samples

**Figure 1:**
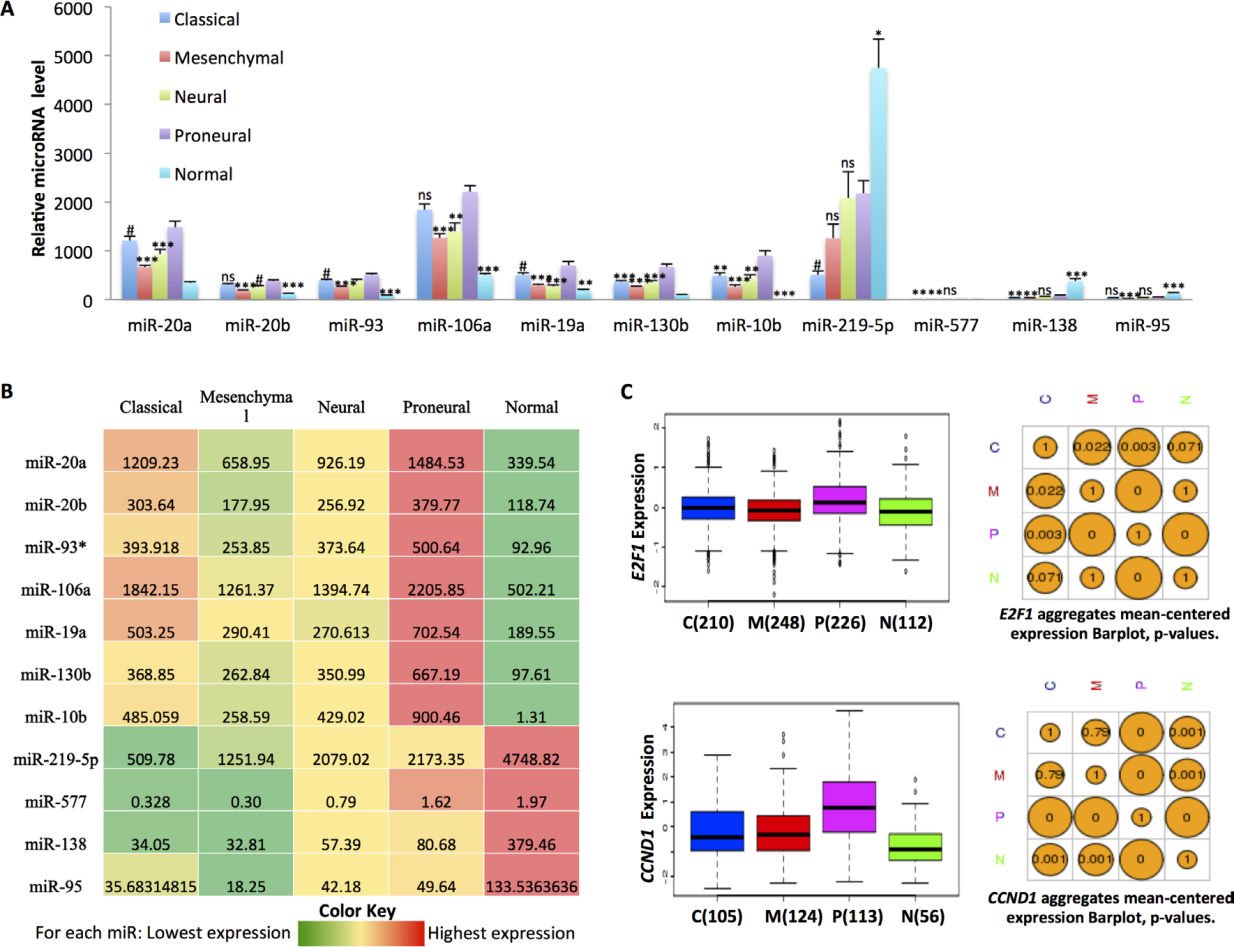
*In silico* analysis of the expression of selected miRNAs from the miR-17˜92 cluster and paralogs shows elevated expression in proneural GBM (**A**) Expression level of miR-17˜92 and paralog miRNAs in the four subtypes of GBM versus normal brain tissues. Classical, *n* = 53; Mesenchymal, *n* = 58; Neural, *n* = 33; Proneural, *n* = 57; Normal, *n* = 10. The difference was analyzed between proneural and the other three subtypes or normal brain tissues. ^#^*p <* 0.05; *p < 0.01; **p < 0.001; ***p < 0.0001; NS, not significant. Data are from TCGA and adapted from Project Betastasis: http://www.betastasis.com/glioma/tcga_gbm. (**B**) Heat map of the miR-17˜92 family expression level in the four subtypes of GBM versus normal brain tissues based on the data shown in panel A. The number denotes the relative expression level of each miRNA in the four subtypes of GBM and normal brain tissues. (**C**) Expression of *E2F1* and *CCND1* in the four subtypes of GBM. C: classical; M: mesenchymal; P: proneural; N: neural. The sample number is shown in the left panel. The *p* values comparing across subtypes are shown in the right panel. Data are adapted from the Glioblastoma Bio Discovery Portal: https://gbm-biodp.nci.nih.gov.

An early report on miRNAs in cancer indicated that miRNA expression patterns could better determine cancer tissue of origin than could gene expression arrays [19]. Similarly, we hypothesized that finding drivers of dysregulated miRNAs provides one approach to identifying core pathways for the GBM subtypes. As a first step toward identifying potential drivers of the GBM subtypes, we did *in silico* analysis of miRNA expression profiles using the TCGA GBM dataset and the previously identified subtyping of these samples [3]. We identified miRNAs with significantly dysregulated expression within each subtype versus others. As shown in Figure 1A and 1B, miR-219-5p, -577, -138, and -95 are down-regulated in all four subtypes compared to normal brain tissues, with the PN subtype exhibiting higher expression of each of these miRNAs. In contrast, miR-20a, -20b, -93, -106a, -19a, -130b, and -10b are up-regulated in GBM compared to the normal brain tissue. Notably, their expression is particularly elevated in PN GBM (Figure 1A, 1B and Supplementary Figure 1). Interestingly, miR-20a and -19a belong to the miR-17˜92 family, while miR-20b and -106a belong to the miR-106a˜363 family, a paralog of the miR-17˜92 cluster [20]. The E2F family of transcription factors and c-myc can bind directly to the promoters of the miR-17˜92 cluster and its paralogs to regulate their transcription [10, 11, 21]. Consistent with a greater role for E2F in PN GBM, E2F1 mRNA is significantly upregulated in the PN subtype versus the other subtypes (Figure 1C). Importantly, the expression levels of other major cell cycle-related genes such as *CCND1, CCNB1*, and *CCNB2* are also significantly elevated in the PN subtype compared to the other subtypes of GBM (Figure 1C and Supplementary Figure 2). Since the E2F pathway drives cell cycle progression and is regulated by CDK4 and CDK6, we postulated that CDK4/6 inhibition would be especially active against PN GBM.

### The miR-17˜92 family and its paralogs are highly expressed in PN GSC lines

**Figure 2:**
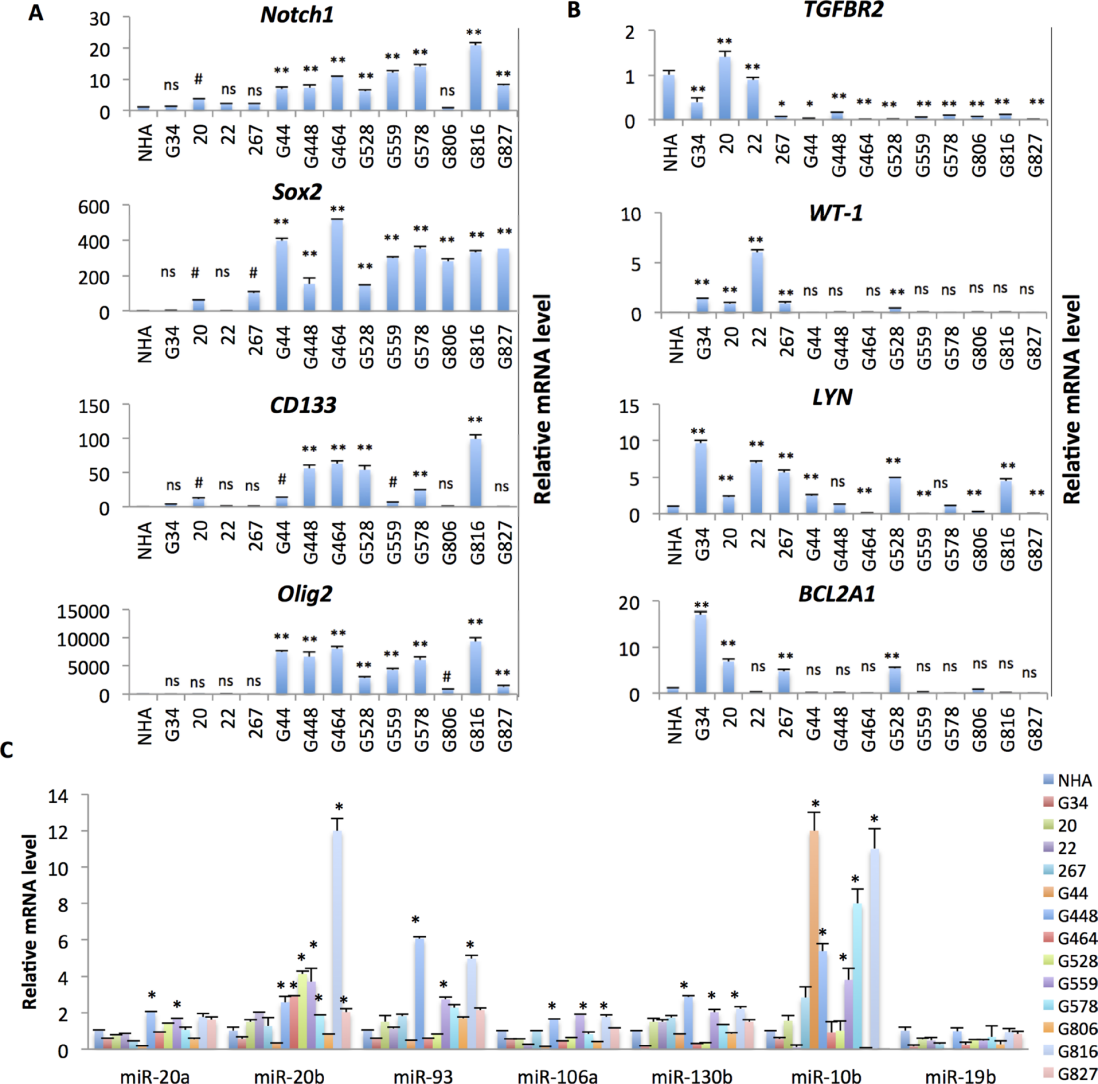
Expression of PN markers and the miR-17˜92 family is elevated in a set of PN GSC lines (**A** and **B**) RT-qPCR analysis of the markers for PN GBM (A, *OLIG22, SOX2, CD133* and *NOTCH1*) and MES GBM (B, *TGFBR2, WT-1, LYN* and *BCL2A1*) in the GSC lines and normal human astrocytes (NHA). *n* = 3. NS, not statistically significant; ^#^*p <* 0.05; *p < 0.01; **p < 0.001; as compared to the expression level in NHA. (**C**) qPCR analysis of the expression of miR-17˜92 family miRNAs and paralogs in the GSC lines and NHA. *n* = 3. *p < 0.01, as compared to the expression level in NHA. Shown are representative data of three independent experiments with similar results.

We next assessed whether the miR-17-92 family and its paralogs are elevated in PN GSC lines. To test this, we utilized a panel of GBM neurosphere lines which were isolated from GBM patient samples and cultured in neural stem cell medium to maintain the original genetic features of the GBM patients [22]. PN and MES GBM are the most well-established subtypes, and may represent opposing ends of a phenotypic axis in GBM [22–24]. To investigate whether the GSC lines display these two distinct expression patterns, we performed qPCR to compare the expression of signature genes of the PN and MES subtypes. As shown in Figure 2A and 2B, one group (8 GSC lines: G44, 448, 464, 559, 578, 806, 816 and 827) showed higher expression of what are considered PN-associated genes (*SOX2, OLIG2, CD133* and *NOTCH1*) and the other (4 GSC lines: G34, 20, 22, and 267) showed higher expression of MES-associated genes (*WT-1, LYN, TGFBR2* and *BCL2A1*). Since some MES markers (*WT-1, LYN and BCL2A1*) are relatively higher, but the PN markers (*SOX2, OLIG2, CD133)* are relative lower in the G528 line, we believe it belongs to the “other” or classical subtype. We then analyzed the expression of the miR-17˜92 cluster and a paralog in the 13 GSCs using qPCR. As predicted, the members of the miR-17˜92 cluster (miR-20a, -20b, -93, -106a, -130b, and -10b) and paralog clusters were over-expressed in seven of eight PN GSC lines (G44, 448, 464, 559, 578, 816 and 827; Figure 2C).

### Most PN GSC lines show greater sensitivity to CDK4/6 inhibitors

**Figure 3:**
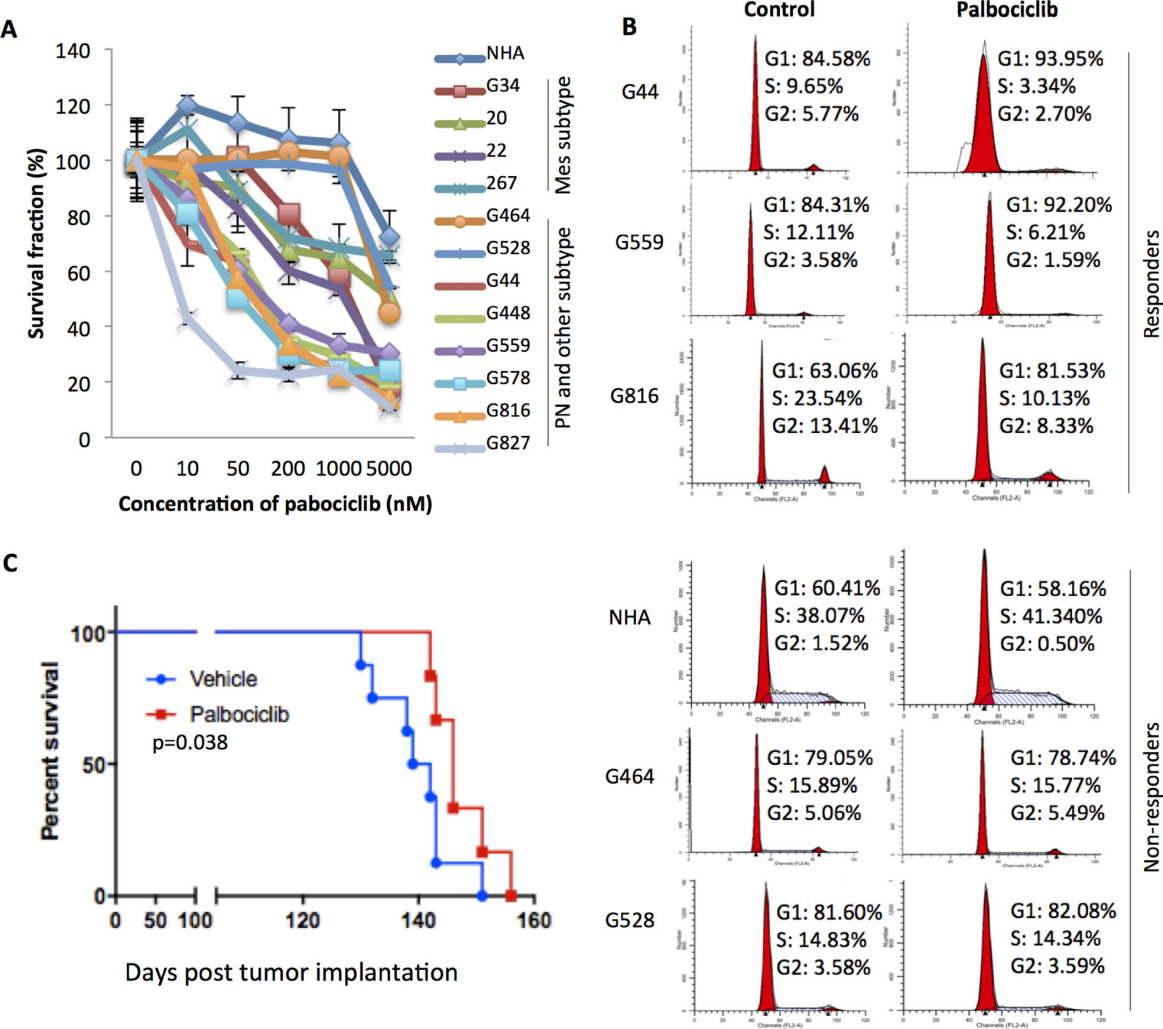
PN GSCs are more sensitive to palbociclib than other GSC subtypes (**A**) Effect of palbociclib on cell proliferation. The twelve GSC lines and normal human astrocytes were seeded at a density of 3,000 cells/well on laminin (10 μg/ml in 0.01% poly-ornithine) coated 96-well plates and treated with the indicated doses of palbociclib for 5 days. *n* = 3. Relative cell number was determined by CyQUANT Direct Cell Proliferation assay. (**B**) Effect of palbociclib on GSC cell cycle distribution. Responder and non-responder lines are shown in left and right panel, respectively. The cells were treated with palbociclib (1 μM) for 5 days prior to the flow cytometry analysis. Shown are representative data of three independent experiments with similar results. (**C**) Kaplan-Meier survival curves of mice after intracranial implantation of G448 (5.0 × 10^5^ cells/mouse) treated with vehicle or palbociclib (150 mg/kg/day, 8 mice/group). The treatment started on day 14 post inoculation and lasted for 4.5 months. Statistical comparisons were performed using Gehan-Breslow-Wilcoxon test. *p* = 0.038.

Given that the E2F cell cycle pathway might play a greater role in PN GBM, we then subjected a panel of twelve GSC lines and a normal human astrocyte (NHA) line to treatment with the CDK4/6 inhibitor palbociclib. The cells were treated with varying concentrations of palbociclib or vehicle for five days, and then assayed for cell number by the CyQUANT Direct Cell Proliferation assay (which measures cell number based on both DNA content and membrane integrity, but is independent of the metabolic state of cells) (Figure 3A). IC50 values for individual cell lines were also determined (Table 1). NHA are highly resistant to palbociclib, with an IC50 of greater than 5 μM. All four MES lines were relatively resistant to the CDK4/6 inhibitor, and the IC50s are equal to or greater than 1 μM. In contrast, six of seven PN GSC lines (G44, 448, 559, 578, 816 and 827) were extremely sensitive to the inhibitors, with IC50s less than 210 nM. Interestingly, one PN line (G464) and one other subtype line (G528) showed stronger drug resistance, exhibiting IC50 values greater than 4.5 μM (Figure 3A, Table 1). Similar dose-response curves were obtained from these PN lines and G528 treated with LEE001, a second CDK4/6 inhibitor (Supplementary Figure 3).

**Table 1:** IC50s and molecular features of the GSCs

weGSC	Subtype	IC50(nM)	CDKN2A(p16)	CDKN2B(p15)	CDKN2C(p18)	CDKN2D(p19)	CDK4	CDK6	RB1
**G34**	MES	1000	−	−	+	+	+	+	+
**20**	MES	5000	+	+	+	+	+	−	−
**22**	MES	1000	−	+	+	+	+	+	−
**267**	MES	> 5000	−	+	+	+	+	+	+
**G44**	PN	210	−	−	+	+	+	++	+
**G448**	PN	100	−	−	+	+	+	++	+
**G464#**	PN	4500	+	+	+	+	+++	+	−
**G528#**	Other	> 5000	+	−	+	+	+++	−	−
**G559**	PN	100	−	−	+	+	+	+++	+
**G578**	PN	50	+	−	+	+	+	++	+
**G816**	PN	80	−	−	+	+	+	+++	+
**G827**	PN	9	−	−	+	+	+	+	−

The IC50 of palbociclib is based on the cell survival results shown on Figure 3A. # indicates the two resistant lines; –, negative expression; +, positive expression. Number of + indicates relative expression level.

The impact of palbociclib treatment on cell cycle distribution was also analyzed using flow cytometry. In the most sensitive PN lines, palbociclib induced accumulation of cells in G1 phase (Figure 3B). In contrast, CDK4/6 inhibition failed to induce G1 arrest in the three resistant lines, NHA, G464, or G528 (Figure 3B). Moreover, daily oral palbociclib treatment (150 mg/kg) significantly increased survival in mice with established intracranial xenografts of the PN GSC line G448, one of the few PN lines showing tumorigenicity *in vivo* (Figure 3C). Taken together, GSC lines exhibited differential sensitivity to the CDK4/6 inhibitor, with most PN GSCs more sensitive to palbociclib than GSCs of other subtypes.

### Molecular abnormalities in the E2F cell cycle pathway in GSC lines

**Figure 4:**
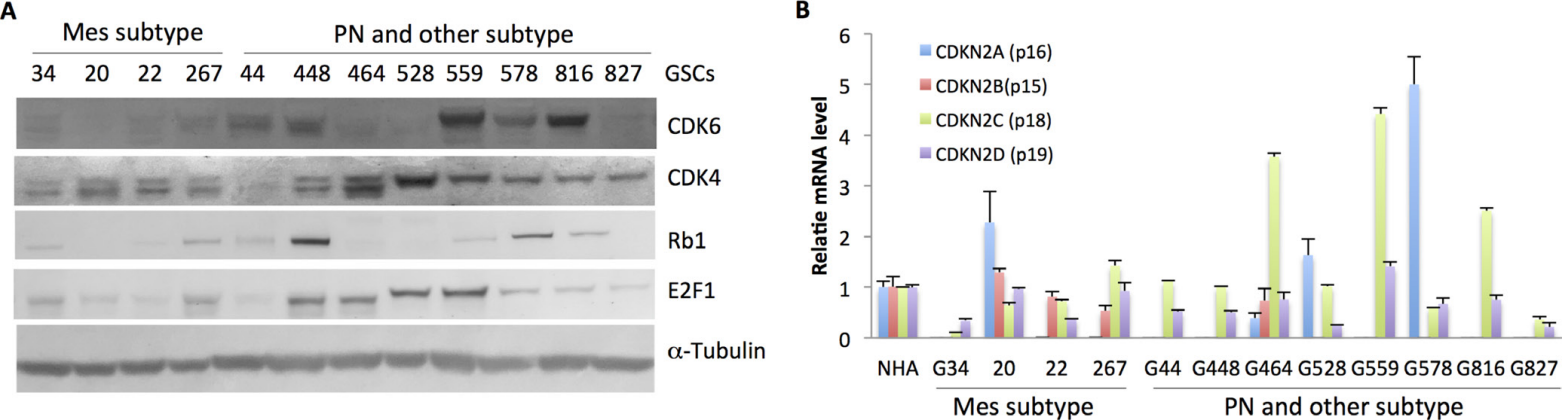
Molecular features of the GSC lines suggest associations with sensitivity and resistance (**A**) Immunoblot analysis of CDK4, CDK6, Rb1, and E2F1 in the GSC lines. α-Tubulin was used as a loading control. (**B**) qPCR analysis of *p15, p16, p18*, and *p19* expression levels in the GSC lines and NHA.

We next explored the mechanisms underlying the differential response to CDK4/6 inhibition across the GSC lines. To this end, we investigated selected components of the E2F cell cycle pathway in the twelve GSC lines using qPCR and immunoblot. Of the six sensitive PN GSC lines, five expressed higher levels of CDK6, whereas all four MES lines expressed lower levels of CDK6. Interestingly, the two most resistant lines (G464 and G528) displayed elevated expression of CDK4 (Figure 4A). Since Rb is tightly regulated by CDK4 and 6 and their cyclin D binding partners, as well as the inactivating CDK inhibitors such as CDKN2A(p16^INK4A^), CDKN2B(p15^INK4B^), CDKN2C(p18^INK4C^), and CDKN2D(p19^INK4D^), we also examined the expression pattern of the four CDK inhibitors. Expression of *p16^INK4A^* was only detected in four of twelve GSC lines (20, G464, G528 and G578), and *p15^INK4B^* was present only in 20, 22, 267 and G464 cells. In contrast, *p18^INK4C^* and *p19^INK4D^* are expressed in all the GSC lines (Figure 4B). Rb1 is present in five of the six sensitive PN GSC lines, with G827 the exception. Of note, Rb1 protein appears absent in the two most resistant GSC lines, G464 and G528. Rb1 protein was present in some of the resistant MES lines, such as G34, 20 and 267 (Figure 4A), indicating that factors other than Rb1 status may determine palbociclib response in some GSC lines—adding to the primary mechanism of palbociclib resistance reported previously [25–27]. The molecular features of the GSC lines are summarized in Table 1. In summary, it appears that the sensitive PN lines express higher levels of CDK6 with intact Rb1, and the resistant PN lines express higher level of CDK4 without Rb1.

### Palbociclib decreases Rb1 phosphorylation and reduces miR-17˜92 family and paralog expression in the sensitive PN GSC lines

**Figure 5:**
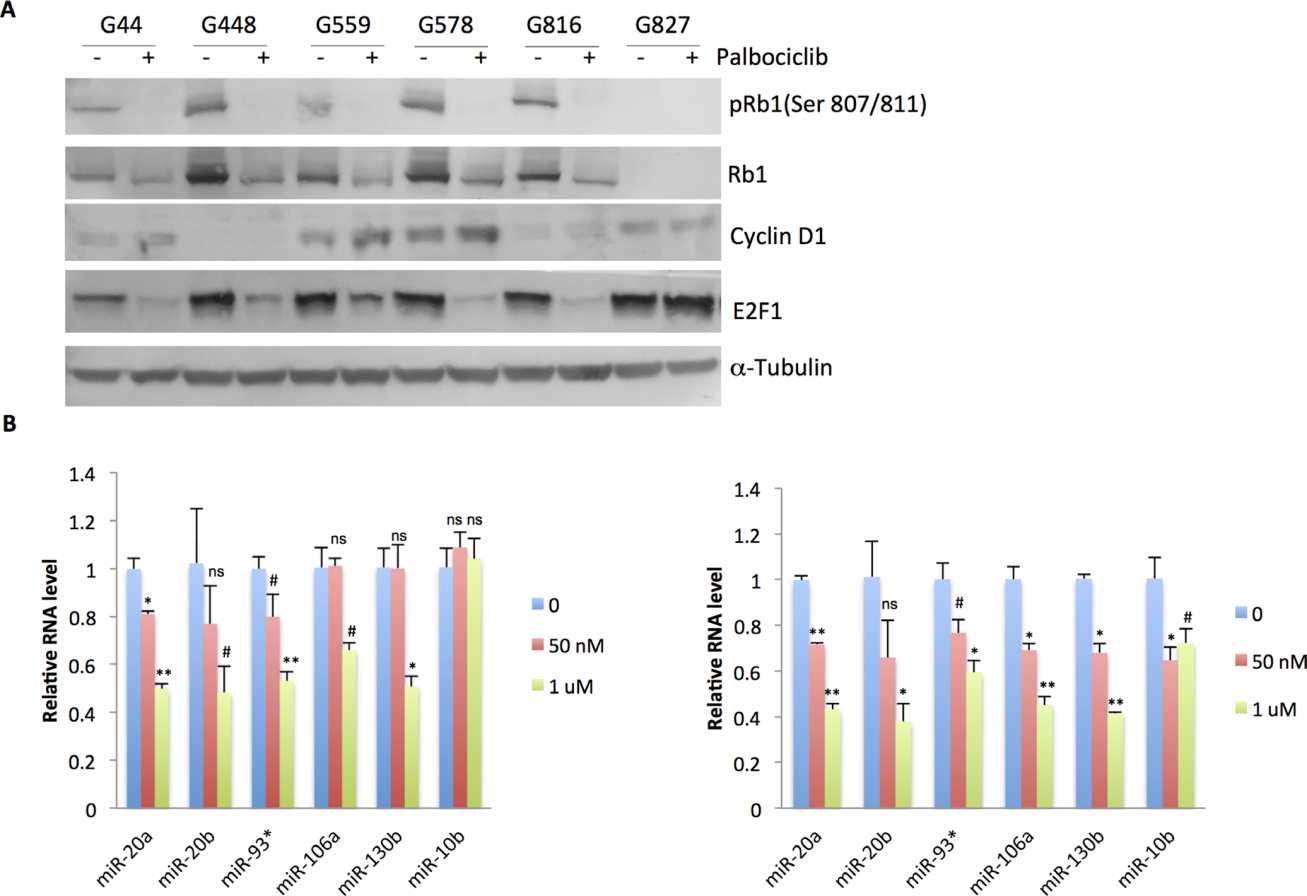
Palbociclib reduces Rb1 phosphorylation and miR17˜92 and paralog expression in the responder PN GSCs (**A**) Immunoblot analysis of Rb1, phospho-Rb1 (Ser 807/811), Cyclin D1, and E2F1 in the palbociclib (1 μM, 24 hr) -treated GSCs. α-Tubulin was used as loading control. (**B**) qPCR analysis of miR-17˜92 and paralog expression in G44 (left panel) and G559 (right panel) treated with the indicated doses of palbociclib for 5 days. *n* = 3. Differences were analyzed between the control and the palbociclib-treated groups. NS: not statistically significant; ^#^*p* < 0.05; *p < 0.01: **p < 0.001. Shown are representative data of three independent experiments with similar results.

CDK4/6-Cyclin D-mediated Rb phosphorylation drives cell cycle progression by releasing E2F transcription factors [28]. Therefore, immunoblot was conducted to examine the effects of palbociclib on Rb1, E2F1, and Cyclin D1 protein expression. In five of the six sensitive PN lines (G44, 448, 559, 578, and 816), palbociclib treatment for 1 day or 5 days dramatically suppressed total Rb1 protein as well as phospho-Rb1 (Ser 807/811) levels. Expression of E2F1, the key target of Rb, was also reduced upon palbociclib administration (Figure 5A and Supplementary Figure 2). Cyclin D1 level was increased in G44, 559, and 578 lines, which is in line with previous reports in other cell types [31–34]. In contrast, in the Rb1-deficient PN line, G827, no obvious changes in the expression levels of E2F1 and Cyclin D1 were detected upon palbociclib treatment (Figure 5A, Supplementary Figure 4).

As described earlier, E2F1 drives expression of the miR-17˜92 family and its paralogs, which are highly expressed in PN GSCs. We examined if palbociclib is able to affect expression of these miRNAs using qPCR. As expected, the CDK4/6 inhibitor significantly decreased the expression of miRNAs in these clusters, including miR-20a, -20b, -93*, and -106a in proneural GSCs (G44 and G559; Figure 5B). Interestingly, palbociclib also reduced expression of two other miRNAs elevated in PN GBM, miR-130b (in both G44 and G559 PN GSC lines) and miR-10b (in G559 PN GSC line).

### Palbociclib may induce a proneural-mesenchymal transition that can be addressed with addition of a MES-selective agent

**Figure 6:**
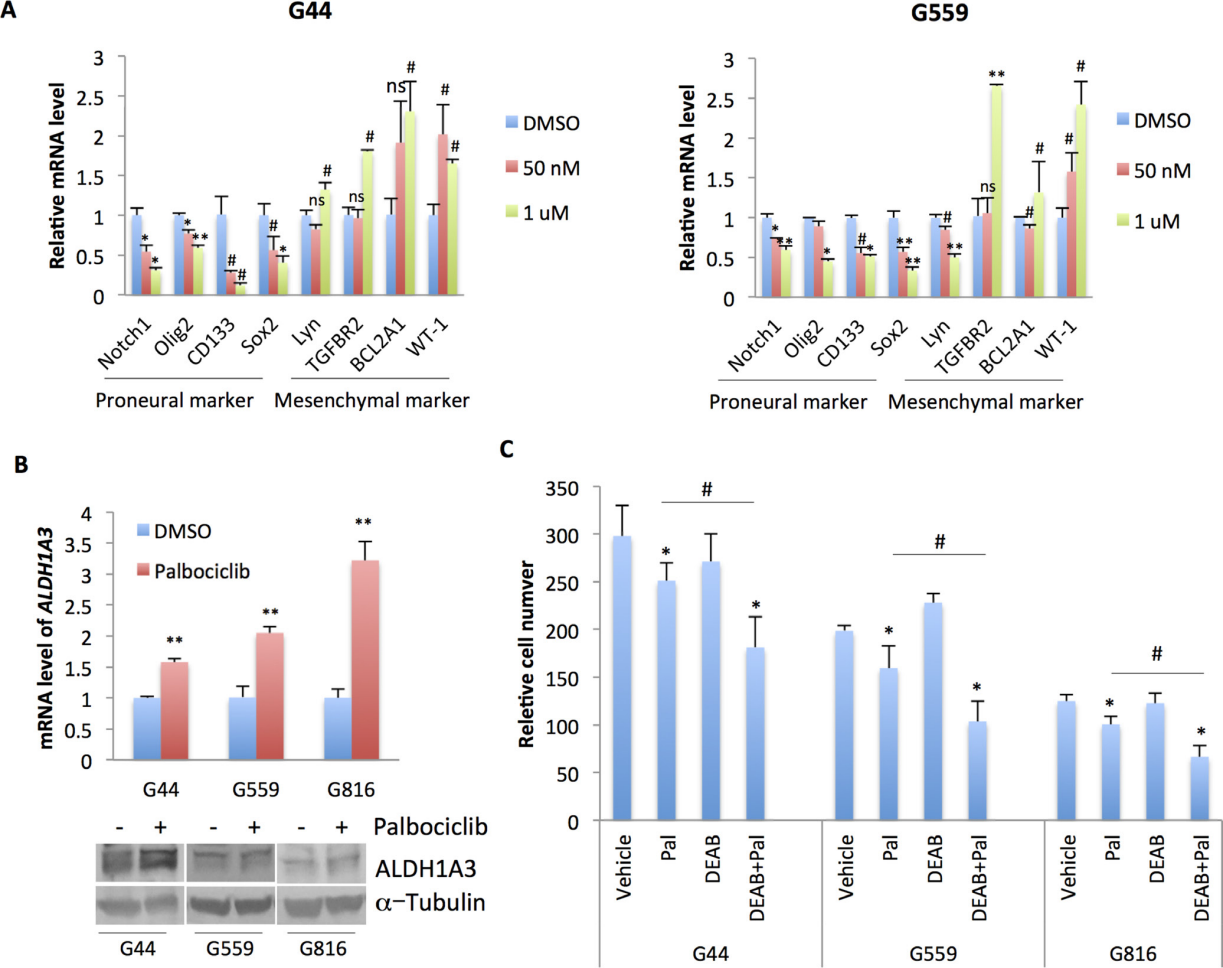
Palbociclib induces a potential transition of PN GSCs to MES GSCs (**A**) qPCR analysis of markers of PN and MES subtypes in G44 and G559 treated with the indicated doses of palbociclib for 5 days. *n* = 3. Differences were analyzed between the control and the palbociclib-treated groups. NS: not statistically significant; #*p* < 0.05; *p < 0.01; **p < 0.001. (**B**) qPCR and immunoblot analysis of ALDH1A3 in three independent PN GSC lines that were treated with 10 nM of palbociclib for 5 days. *n* = 3. Differences were analyzed between the control and the palbociclib-treated groups. NS: not statistically significant; ^#^*p* < 0.05; *p < 0.01; **p < 0.001. (**C**) Cell proliferation analysis of three independent PN GSC lines that were treated with palbociclib (10 nM), DEAB (25 μM), or both for 5 days. The cells were cultured on a laminin (10 μg/ml in poly ornithine)-coated 96-well plate. Cell number was determined by CyQUANT Direct Cell Proliferation assay. *n* = 3. *p < 0.05, comparison between palbociclib or DEAB treatment with the vehicle control; ^#^*p <* 0.05, comparison between combination group with palbociclib treatment. G44, CDI = 0.79; G559, CDI = 0.565; G816, CDI = 0.677. Shown are representative data of three independent experiments with similar results.

Palbociclib has been shown to be involved in an Epithelial-Mesenchymal transition (EMT) in pancreatic and breast cancer [29, 33]. Given these findings in other cancers and our demonstration of proneural sensitivity to palbociclib, we hypothesized that CDK4/6 inhibition might trigger a shift in GSC subtype. We performed RT-qPCR and found that palbociclib treatment of PN GSC lines decreased expression of 4 PN markers (*NOTCH1, OLIG2, CD133* and *SOX2*), while expression of the MES markers *TGFBR2, BCL2A1*, and *WT-1* increased (Figure 6A) —suggesting a possible PN-MES transition (PMT) in palbociclib-treated PN GSCs.

Since ALDH1A3 has been recently shown to be aberrantly up-regulated in MES GSCs compared with PN GSCs [22], we examined whether its expression is affected by CDK4/6 inhibition. A low dose of palbociclib (10 nM) significantly up-regulated *ALDH1A3* mRNA expression in PN GSC lines (*p* < 0.01, Figure 6B). We then examined if an ALDH1A3 inhibitor, DEAB, might be able to oppose the prospective PN-MES transition and act synergistically with palbociclib. DEAB (25 μM) alone had no obvious effect on the proliferation of PN GSC lines. However, when it was combined with palbociclib (10 nM) to treat PN GSCs, cell proliferation was significantly inhibited compared to the cells treated with palbociclib or DEAB alone (For G44, CDI = 0.79; for G559, CDI = 0.565; for G816, CDI = 0.677; Figure 6C). Taken together, these data suggest that palbociclib treatment of PN GSCs induces a possible PN-MES transition that can potentially be ameliorated with the addition of an agent with reported MES-selective activity.

## DISCUSSION

While previous reports have linked certain microRNA profiles to particular GBM subtypes [34–36], to our knowledge this is the first example of leveraging subtype-overexpressed miRNAs to identify underlying driver pathways. While studies of gene expression have been the focus of past studies to identify upstream core transcriptional drivers of subtypes in GBM and other cancers, the far smaller number of miRNAs [37] may facilitate such efforts. Here, our *in silico* analysis showed the miR-17˜92 cluster and its paralogs to be elevated in PN GBM, potentially indicating an enhanced role for the E2F cell cycle pathway and potential sensitivity to a CDK4/6 inhibitor in PN GBM. Hints in prior reports also supported this hypothesis. One report demonstrated that the miR-17˜92 cluster targets TGF-β signaling, a potential driver of mesenchymal GBM, and therefore over-expression of this miRNA cluster should push cancer cells toward a proneural or epithelial phenotype [38]. A more recent study showed that platelet-derived growth factor (PDGF), known to play a significant role in proneural GBM, drives E2F-USP1 signaling in proneural glioma [39].

It has been reported that Rb status is the primary determinant of sensitivity to CDK4/6 inhibition [40]. In breast cancer, Rb1 deficiency or loss of its function results in palbociclib resistance, and the same has been shown for GBM [30, 40–45]. In line with these results, one report has shown that changes in the E2F-Rb1 cell cycle pathway, including higher levels of Cyclin D1 and Rb1 and lower level of p16, led to the greatest sensitivity to palbociclib treatment [46]. Another report has shown that co-deletion of *p16* and *p18* predicts palbociclib sensitivity, and that higher levels of *CDK4* or *CDK6* had no influence on sensitivity [44]. However, this was not the case in our study, in which we found p18 to be present in all 12 GSC lines and p16 to be detectable in the two most resistant lines (G464 and G528). In contrast, in line with a recent report [45], our results suggest that CDK4 overexpression may be a marker for palbociclib resistance even in proneural GBM, while CDK6 over-expression may be associated with sensitivity. Specifically, nearly all of the sensitive PN GSC lines express higher level of CDK6 and have Rb1 protein expression, while p15 and p16 are absent in these cells.

It has been noted that palbociclib may also have an Rb1-independent anti-proliferative effect. Daniel *et al*. recently observed similar responses to palbociclib in *Rb1*-wild-type and *Rb1*-mutant bladder cell lines *in vitro* and in xenografts *in vivo* [25]. Similarly, palbociclib has proven activity in Rb1-deficient prostate cancer cells [26] and hepatocellular carcinoma cells *in vitro*, in which some activity in Rb1-deficient cells may be compensated by related proteins such as p107 [27]. Interestingly, in our study an Rb1-deficient PN GSC line (G827) displayed the greatest sensitivity to palbociclib treatment. While our study and others suggest additional markers for palbociclib sensitivity, further work on this important area is needed.

Inhibition of Rb1 phosphorylation is a downstream mediator of CDK4/6 inhibition. We showed that palbociclib decreased total Rb1 levels as well as Rb1 phosphorylation, likely due to enhanced Rb1 degradation. We also observed that palbociclib increased Cyclin D1 levels in half of the sensitive GSC lines. As has been suggested by Dean *et al*. [30], Cyclin D1 stability is likely increased through proximal effects on the CDK4/6–Cyclin D1 complex, and the elevated Cyclin D1 could fuel rapid activation of CDK4/6 if the levels of palbociclib were to become limiting—suggesting that continual treatment might be needed. Notably, elevated Cyclin D1 is implicated in both EMT and cell senescence [31–32, 47]. Interestingly, CDK4/6 inhibition also decreased E2F1 expression in the Rb1-intact sensitive GSCs, but had no obvious effect on E2F1 protein expression in the Rb1-deficient line G827. Since it had a similar anti-growth effect in these lines, this may indicate that other effectors are also involved in this process.

These results indicated that CDK4/6 inhibition in PN GBM decreased expression of PN markers and increased MES marker expression, indicating there might be a shift from PN to MES subtype. A similar phenomenon has been observed in pancreatic cancer models, in which palbociclib treatment prompted an EMT response [29]. However, Qin *et al*. reported that palbociclib inhibits EMT in breast cancer [33], indicating that EMT induced by CDK4/6 inhibition might be cell type-dependent. While a treatment-induced PN-MES shift in GBM has been proposed in other reports [3, 48], the rapidity of it in these experiments was remarkable. This prompted us to seek an answer to what seems to be an acute resistance mechanism. We found that a low concentration of palbociclib was able to induce the expression of ALDH1A3, previously shown to be a MES marker and potential driver in GBM, and which plays a role in the initiation and progression of tumors via the clearance of aldehydes and the production of retinoic acid [22]. Importantly, combination of the ALDH1A3 inhibitor, DEAB, with palbociclib synergistically inhibited proneural GSC cell proliferation.

In summary, we show that miRNA expression profile analysis may provide new insights into the druggable drivers of cancer subtypes such as PN GBM. CDK4/6 inhibition has an enhanced anti-proliferative effect against most PN GSCs, and a rapid PN-MES transition may occur as a protective mechanism. We propose that palbociclib may best be applied against PN-predominant GBM, and that combination with a MES-selective agent such as DEAB may help block resistance. These findings may also have relevance for other cancers, given that the proneural-mesenchymal axis in GBM seems to parallel the epithelial-mesenchymal axis in many epithelial malignancies.

## MATERIALS AND METHODS

### Cell culture and reagents

GSC lines were obtained from Jeongwu Lee (Cleveland Clinic), Jakub Godlewski and Agnieszka Bronisz at Brigham and Women's Hospital, Ichiro Nakano (University of Alabama), and Krishna Bhat and Erik Sulman (MD Anderson Cancer Center) and were derived as previously described [6]. The GSC lines were cultured in Neurobasal medium supplemented with N-2 (Invitrogen), B27 (Invitrogen), glutamine, EGF (25 ng/ml) and bFGF (25 ng/ml) to maintain the stemness and genetic features of the clinical sample. When necessary, the GSC lines were cultured in the above stem cell medium on tissue culture plates coated with laminin (Roche, 10 μg/m in 0.01% poly-ornithine, Sigma). This laminin monolayer culture condition was previously reported to be equivalent to neurosphere culture for glioblastoma stem cell-like lines [16, 17]. Human origin of GBM stem cell lines was confirmed by short tandem repeat analysis within the last six months.

Immortalized normal human astrocytes (NHA) were maintained in DMEM with 10% FBS. Palbociclib and LEE001 (ribociclib) were purchased from Selleckchem. N,N-diethylaminobenzaldehyde (DEAB) was obtained from Sigma.

### *In silico* gene and miRNA expression analysis

The expression profiles of miRNAs and genes were analyzed using the online TCGA GBM dataset: 1) Project Betastasis: http://www.betastasis.com/glioma/tcga_gbm; 2) The Glioblastoma Bio Discovery Portal: https://gbm-biodp.nci.nih.gov.

### Western blots and antibodies

Cells were lysed in RIPA buffer (Cell Signaling Technology) with complete protease and phosphatase inhibitor (Roche). Primary antibodies used for Western blot were: Rb1 (9309), phopho-Rb1 (Ser807/811) (9308), Cyclin D1 (2922), CDK6 (3136), CDK4 (12790), and E2F1 (3742) (Cell Signaling Technology), ALDH1A3 (NBP2-15339, Novus Biologicals), and α-Tubulin (Sigma). Secondary anti-Rabbit IgG and anti-mouse IgG were purchased from Sigma.

### Cell proliferation assay

Cell viability was assessed using the CyQUANT Direct Cell Proliferation Kit (ThermoFisher Scientific). Cells were plated in triplicate onto a laminin (10 μg/ml in 0.01% poly-ornithine) coated 96-well plate at a concentration of 3,000 cells/well and began treatment the next day with palbociclib or vehicle for 5 days. The detection reagent was added to wells at a 1:1 ratio and incubated at 37°C for 1 hr. Fluorescence was measured at 480/535 nm using GENios Pro (Tecan).

### Flow cytometry analysis

Cell cycle distribution was analyzed on BD Facscalibur at the Flow Core of the University of Virginia. The palbociclib-treated cells were collected, washed with PBS, and fixed in ice-cold 70% ethanol/10% PBS for at least 2 hr. The fixed cells were then centrifuged for 5 min at 300 g, resuspended in 1 ml propidium iodide (PI) staining solution with 100 μg/ml RNase A and 0.05% Triton X-100, and incubated for 30 min at 37°C. The results were analyzed using Modfit software.

### qPCR

Total RNA was harvested by QIAzol reagent (Qiagen) and reverse-transcribed (SuperScript III First Strand kit; Invitrogen). For gene expression analysis, qPCR was performed with 2 μl of diluted cDNA on an Applied Biosystems StepOnePlus PCR machine using Power SYBR Green (Applied Biosystems) according to the manufacturer's instructions. All reactions were performed in triplicate and repeated at least two times. Relative quantification was performed for each sample and normalized with GAPDH expression for comparison. Primers used for qPCR were listed in Supplementary Table 1. Gene expression levels were calculated using the relative Δ*C*t method.

For miRNA expression analysis, single-stranded cDNAs from total RNA samples were synthesized using the TaqMan MicroRNA Reverse Transcription Kit (Applied Biosystems). Mature miRNA expression was determined using small RNA TaqMan assays according to the manufacturer's instructions (Applied Biosystems). All reactions were done in triplicate. The expression of miRNAs was normalized using RNU48. The expression relative to RNU48 was determined using the Δ*C*t method.

### Xenograft model

All animal experiments conformed to ethical principles and guidelines approved by the Institutional Animal Care and Use Committee at the University of Virginia. 5.0 × 10^5^ of G448 cells in 5 μl PBS were injected intracranially into 6–8 week old SCID Ncr mice. Beginning 14 days after surgery, 100 μl of palbociclib (150 mg/Kg in water) or water was given daily via oral gavage (8 mice/group) [37]. The survival and health of the tumor-bearing mice were closely monitored. Survival data were plotted on a Kaplan–Meier curve. Statistical comparisons were performed using Gehan-Breslow-Wilcoxon test.

### Statistical analysis

Data are expressed as the mean ± SD. Statistical analyses were performed using GraphPad Prism 7.00. Student's *t* test was used to compare the difference between the control and the treatment group. One-way ANOVA was used to compare the difference between multiple groups. *P* < 0.05 was considered statistically significant.

The coefficient of drug interaction (CDI) was used to analyze drug combinatorial effects [18]. CDI is calculated as follows: CDI = AB/(A × B). AB is the ratio of the combination groups to the control group; A or B is the ratio of the single agent groups to the control group. CDI < 1 indicates synergism, and CDI < 0.7 indicates a significantly synergistic effect; CDI = 1 indicates additivity; and CDI > 1 indicates antagonism.

## SUPPLEMENTARY MATERIALS FIGURES AND TABLE


