# Supplementary material for: CDK4/6 inhibition is more active against the glioblastoma proneural subtype
Source: Oncotarget. 2017 Jul 21;8(33):55319–31. doi: 10.18632/oncotarget.19429 (PMC5589661; doi:10.18632/oncotarget.19429)
Supplement: Supplementary file 1 [file oncotarget-08-55319-s001.pdf]

CDK4/6 inhibition is more active against the glioblastoma proneural subtype

SUPPLEMENTARY MATERIALS

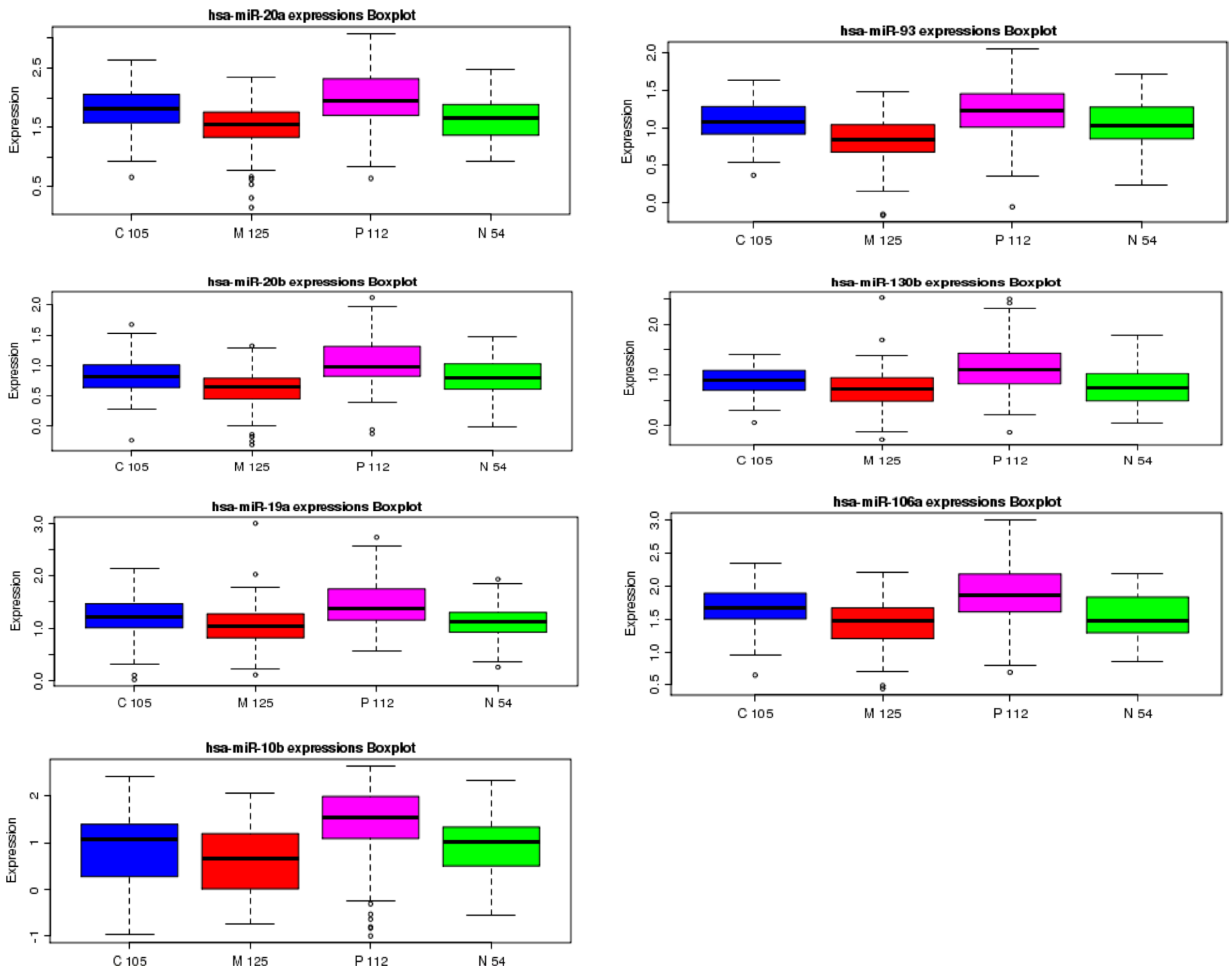

Supplementary Figure 1: Expression level of miR-17~92 and paralog miRNAs in the four subtypes of GBM. *P* values are less than 0.01 when comparing the level of each miRNA in the PN subtype versus the other three subtypes. Data are adapted from the Glioblastoma Bio Discovery Portal: https://gbm-biodp.nci.nih.gov.

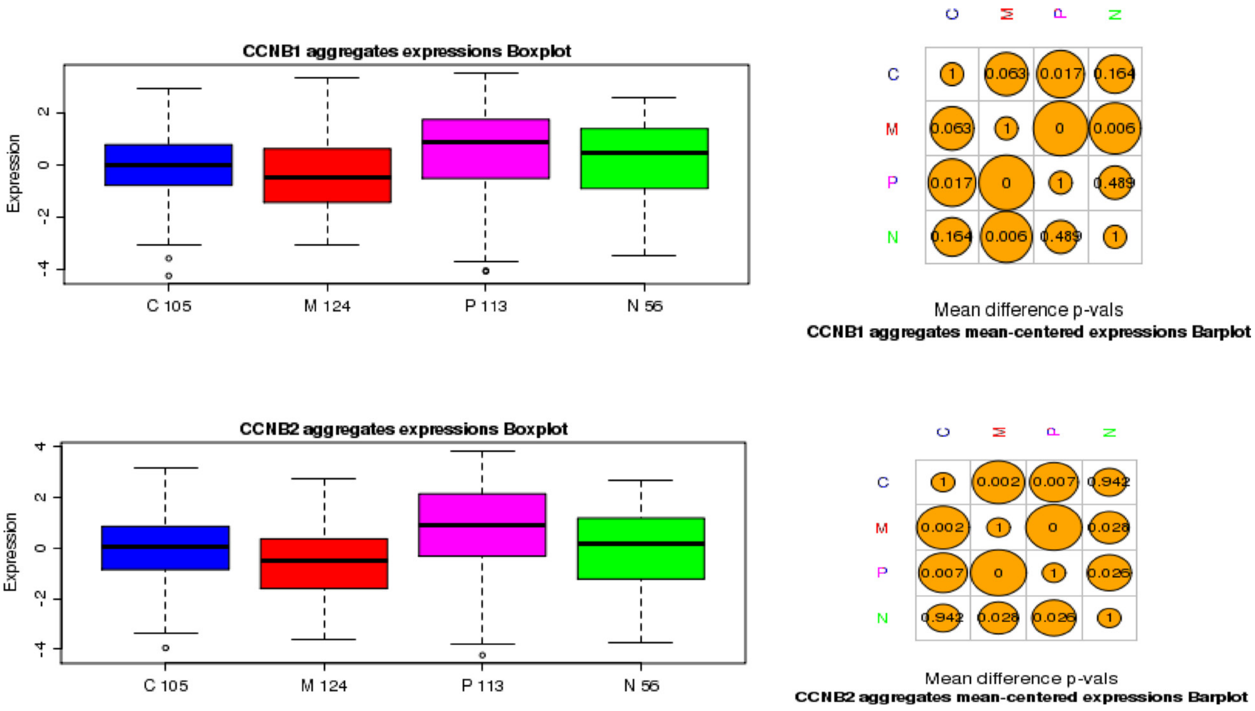

Supplementary Figure 2: Expression levels of *CCNB1* and *CCNB2* in the four subtypes of GBM. C: classical; M: mesenchymal; P: proneural; N: neural. Sample number is shown in the left panel. The *p* values for comparisons across subtypes are shown in the right panel. Data are adapted from the Glioblastoma Bio Discovery Portal: https://gbm-biodp.nci.nih.gov.

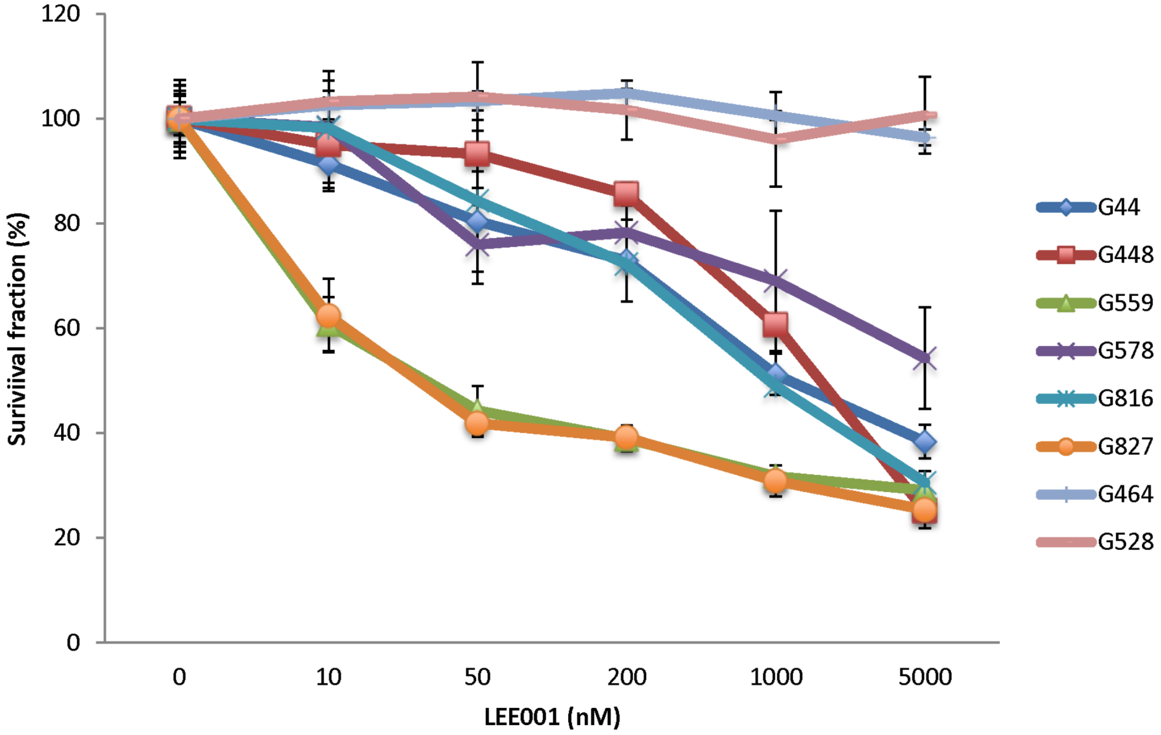

Supplementary Figure 3: Effect of LEE001 on GSC cell proliferation. Seven PN GSC lines and the G528 line were seeded at a density of 3,000 cells/well on laminin-coated (10 µg/ml in 0.01% poly-ornithine) 96-well plates and treated with the indicated doses of LEE001 for 5 days. *n* = 3. Relative cell number was determined by CyQUANT Direct Cell Proliferation assay.

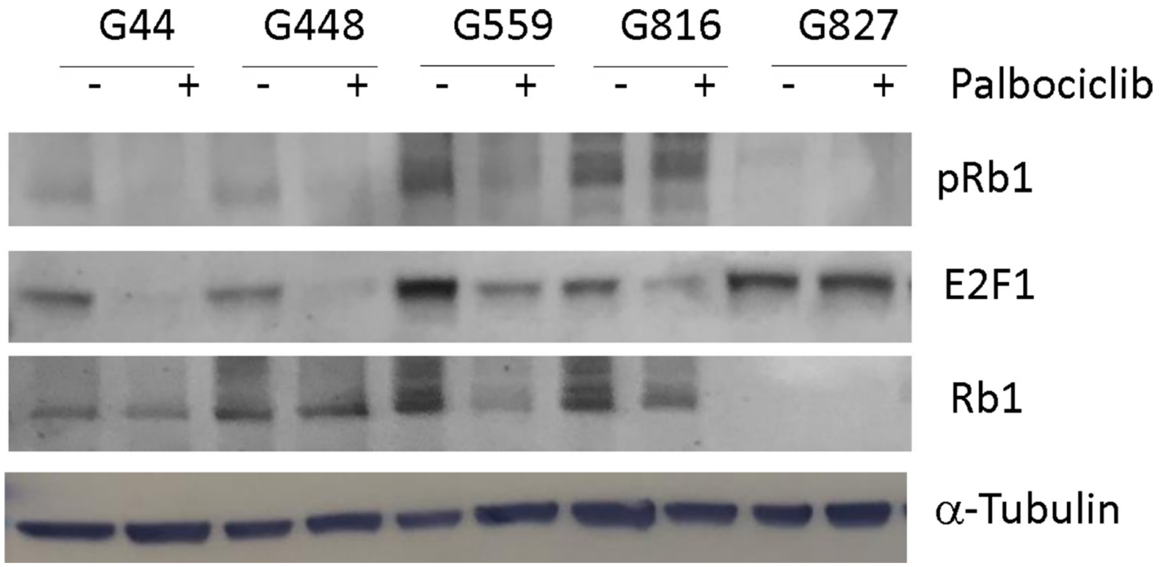

Supplementary Figure 4: Palbociclib reduces Rb1 and E2F1 protein level in PN GSC lines. The protein level was examined by immunoblot in the indicated cells treated with 1 µM of palbociclib for 5 days.

Supplementary Table 1: List of primer sequences used for RT-qPCR analysis in this study

Genes	Primer sequences (5′—3′)
GAPDH	F: GAAGGTGAAGGTCGGAGTCA<br>R: TTGAGGTCAATGAAGGGGTC
CD133	F: ACTCCCATAAAGCTGGACCC<br>R: TCAATTTTGGATTCATATGCCTT
OLIG2	F: CTCCTCAAATCGCATCCAGA<br>R: AGAAAAAGGTCATCGGGCTC
SOX2	F: ACCGGCGGCAACCAGAAGAACAG<br>R: GCGCCGCGGCCGGTATTTAT
NOTCH1	F: AGTGTGAAGCGGCCAATG<br>R: ATAGTCTGCCACGCCTCTG
LYN	F: CTGAACTCAAGTCACCGTGG<br>R: TCCATCGTCACTCAAGCTGT
WT-1	F: TTAAAGGGAGTTGCTGCTGG<br>R: GACACCGTGCGTGTGTATTC
BCL2A1	F: ATGGATAAGGCAAAACGGAG<br>R: TGGAGTGTCCTTTCTGGTCA
TGFBR2	F: CTGCACATCGTCCTGTGG<br>R: GGAAACTTGACTGCACCGTT
CDK6	F: TGGAGACCTTCGAGCACC<br>R: CACTCCAGGCTCTGGAACTT
CDK4	F: TGCAGTCCACATATGCAACA<br>R: GTCGGCTTCAGAGTTTCCAC
p15	F: TACAGGAGTCTCCGTTGGC<br>R: GTGAGAGTGGCAGGGTCTG
p16	F: GTTACGGTCGGAGGCCG<br>R: GTGAGAGTGGCGGGGTC
p18	F: ACGTCAATGCACAAAATGGA<br>R: CAAATCGGGATTAGCACCTC
p19	F: AACCGCTTCGGCAAGAC<br>R: GTCCTGGACATTGGGGCT
ALDH1A3	F: TGGATCAACTGCTACAACGC<br>R: CACTTCTGTGTATTCGGCCA

F: forward primer; R: reverse primer.
